# Multimodal Biomedical Image Segmentation using Multi-Dimensional U-Convolutional Neural Network

**DOI:** 10.1186/s12880-024-01197-5

**Published:** 2024-02-08

**Authors:** Saravanan Srinivasan, Kirubha Durairaju, K. Deeba, Sandeep Kumar Mathivanan, P. Karthikeyan, Mohd Asif Shah

**Affiliations:** 1 Department of Computer Science and Engineering, Vel Tech Rangarajan Dr.Sagunthala R&D Institute of Science and Technology, Avadi, Chennai, India, Chennai, India; 2 Department of Computer Science and Engineering, Rajarajeswari College of Engineering, Bangalore, 560074 India; 3 School of Computer Science and Applications, REVA University, Bangalore, 560064 India; 4 School of Computing Science and Engineering, Galgotias University, Greater Noida, 203201 India; 5 Department of Computer Applications,School of Computer Science Engineering and Information Systems, Vellore Institute of Technology, Vellore, Tamil Nadu 632014 India; 6 Department of Economics, Kabridahar University, Po Box 250, Kabridahar, Ethiopia; 7 Centre of Research Impact and Outcome, Chitkara University Institute of Engineering and Technology, Chitkara University, Rajpura, 140401, Punjab India; 8 Division of Research and Development, Lovely Professional University, Phagwara, Punjab 144001, India

**Keywords:** U-net, Multimodal convolutional neural network, Segmentation, Medical image, MDU-CNN

## Abstract

Deep learning recently achieved advancement in the segmentation of medical images. In this regard, U-Net is the most predominant deep neural network, and its architecture is the most prevalent in the medical imaging society. Experiments conducted on difficult datasets directed us to the conclusion that the traditional U-Net framework appears to be deficient in certain respects, despite its overall excellence in segmenting multimodal medical images. Therefore, we propose several modifications to the existing cutting-edge U-Net model. The technical approach involves applying a Multi-Dimensional U-Convolutional Neural Network to achieve accurate segmentation of multimodal biomedical images, enhancing precision and comprehensiveness in identifying and analyzing structures across diverse imaging modalities. As a result of the enhancements, we propose a novel framework called Multi-Dimensional U-Convolutional Neural Network (MDU-CNN) as a potential successor to the U-Net framework. On a large set of multimodal medical images, we compared our proposed framework, MDU-CNN, to the classical U-Net. There have been small changes in the case of perfect images, and a huge improvement is obtained in the case of difficult images. We tested our model on five distinct datasets, each of which presented unique challenges, and found that it has obtained a better performance of 1.32%, 5.19%, 4.50%, 10.23% and 0.87%, respectively.

## Introduction

Segmenting skin lesions using computer assistance becomes difficult due to variations in their shapes and sizes. CAD methods rely on proper lesion segmentation as a crucial early step to obtain precise evaluations of skin lesion borders and sizes. The majority of expert dermatologists have found the subjective clinical segmentation evaluation of skin lesions using deep learning-based techniques to be insufficient [1]. This study primarily aims to utilize advanced techniques, such as deep learning-based automated skin lesion segmentation, to enhance the accuracy and efficiency of melanoma classification, while dermoscopy images aid medical professionals in detecting melanoma in its initial stages. The U-net algorithm, which depends on convolutional neural networks, serves as an indispensable tool for carrying out the segmentation process. Deriving colour and shape features from the segmented image using a local binary pattern along with an edge histogram is highly effective. Various classifiers, such as random forest and naive Bayes, were used to analyze all the features extracted from the skin images to determine if they contained melanoma or benign lesions [2]. This study aims primarily to leverage state-of-the-art approaches, such as automated skin lesion segmentation that uses deep learning techniques, to boost the precision and swiftness of classifying melanomas while also aiding medical professionals in spotting them at an early stage through dermoscopy images. Additionally, the U-net algorithm, which utilizes a convolutional neural network, is a vital component in performing the segmentation. The combination of a local binary pattern and an edge histogram is highly effective for extracting colour and shape features from the segmented image. The analysis of all the extracted features from skin images was conducted by applying different classifiers, including random forest and naive Bayes, to detect the existence of either melanoma or benign lesions [3]. The worldwide fatality rates attributed to melanoma make it one of the deadliest and most aggressive types of skin cancers. Clinical practitioners typically employ biopsy techniques along with microscopic analyses when screening for or preventing skin cancers. The use of a dermatoscope to capture clear images of the affected area is critical in understanding lesion patterns and aiding diagnosis. Additionally, as the lesion evolves and changes its shape over time, manual segmentation of its region can be tough to predict and quite arduous [4].

Melanoma-related deaths due to skin cancer have significantly surged in recent times. However, survival rates may increase for individuals with this chronic condition if melanoma skin disease lesions are detected early. Identifying these lesions is often challenging as they may be obstructed by clinical objects or appear differently due to variations in colour or contrast. Nevertheless, state-of-the-art techniques for sorting and diagnosing involve the use of fully convolutional neural networks that employ an encoder-decoder technique. The encoding layers in these techniques may result in the loss of location information, thus generating granular segmentation-based masks [5]. The reports of rising death toll each year due to melanoma skin cancer indicate a significant increase in its prevalence. Therefore, creating an efficient and successful non-invasive computerized diagnosis tool that precisely detects melanoma by segmenting skin lesions is imperative. This study addresses the segmentation of melanoma skin lesions in dermoscopic images by introducing a new algorithm that utilizes both perceptual colour distinctions and a binary morphological method [6].

The mortality rate for melanoma is high once it has advanced to that stage. However, scientists have worked hard to design automatic systems that can rapidly recognize this fatal sickness. Due to the wide array of skin lesion volumes and shades present in melanoma moles, the identification process is often a challenging task that consumes substantial amounts of time. The presence of noise or blurring, along with luminance modifications in suspicious images, contributes to making detection more complex. To overcome the limitations of previous research, we demonstrate a DL model in this paper [7]. Excessive sunlight exposure can often be attributed as the cause of melanoma, with digital dermoscopy serving as a tool for identifying cancerous areas within skin lesions. To accurately use automated lesion recognition and classification, one must distinguish between healthy skin tissue and cancerous cells. Lesion segmentation has an impact on both the accuracy and precision of classification. Therefore, the focal point of this study is the introduction of a new lesion classification system. Hair filtration, the use of bubbles, and improving specular perception are some of the means to choose from. An improved technique utilizing advanced levelling methodology has been specifically created for the detection and removal of malignant hair [8]. Treating cancerous cells on pigmented skin lesions by simple ablation after visually examining them allows for the timely identification of melanoma. However, performing several biopsies is necessary because visual exams can have variable accuracy levels when there is a shortage of dermatologists who can perform them. To improve the performance of computerized melanoma segmentation methods in dermoscopic images, this work suggests a deep learning-based approach [9]. Melanoma may be lethal, but if detected at an early stage, it can be treated and cured. However, accurate identification of squamous cell carcinoma versus a benign tumor is essential. For this reason, the use of computer-generated recognition to obtain dermoscopy images has become popular, and automated and accurate classification of melanoma is the primary goal of this study. Analyzing the efficiency of this approach in terms of identifying melanoma is done using the ISBI 2016 skin lesion assessment dataset [10].

## Related work

Shifa Kubra N et al. [11], the researchers explored the capability of deep convolutional neural networks in distinguishing between benign and cancerous skin cells. Their study utilized a sample size of 3600, with 3000 samples used for training and the remaining portion used for validation, specifically focusing on dermoscopy images. The findings indicated that deep learning models outperformed human dermatologists in terms of accuracy. By employing very deep neural networks along with switch reversal and fine-tuning on dermoscopy images, the researchers were able to achieve superior diagnostic capabilities compared to experienced clinicians and oncologists. In another study by Chen Zhao et al. [12], the researchers emphasized the significance of fully connected neural networks and U-Net in the melanoma segmentation process. However, they identified that as the depth of these deep neural networks increased, the issue of gradient vanishing arose, making them susceptible to parametric redundant systems. This issue had a negative impact on skin lesion image segmentation and resulted in a decrease in the Jaccard index value. To address these challenges and improve the survival rate of melanoma patients, the paper proposed an enhanced segmentation process based on U-Net++. This approach aimed to tackle the mentioned issues and enhance the accuracy of melanoma segmentation. Andrea Pennisi et al. [13] aimed to create an AI-based system that assesses relationships between images taken at different points in time. The first step in this process is to divide up the affected part of the lesion. Moreover, to detect the edges of skin lesions in medical images, we propose using an attention squeeze U-net model based on deep learning techniques. Based on the quantitative outcomes obtained from an open-access dataset, it appears feasible to achieve accurate segmentation using a simplified approach.

Nojus Dimša et al. [14], encoder and decoder structures are beneficial for object segmentation, particularly the U-Net framework, which serves as the foundation for segmenting medical images in a network system. Various combinations of U-Net-type layouts have been introduced recently in an effort to improve segmentation outcomes. Thus, we evaluated the ability and effectiveness of three U-Net type models, namely U-Net, U-Net++, and MultiResU-Net, for the multi-class segmentation of melanoma. Lina Liu et al. [15] propose utilizing an advanced deep convolutional neural network framework referred to as the U-Net model to perform accurate segmentation of skin lesions. By introducing batch normalization layers in our modified version of U-Net, along with an enhanced convolutional neural network architecture, we were able to prevent prediction errors and enrich the perceptron during the training phase. Results of experimental evaluation have demonstrated that adding enlarged convolution can considerably enhance the effectiveness of the presented method. Additionally, we present a simple, direct, yet practical experimental ensemble approach that does not require training additional frameworks.

Haoran Lu et al. [16] demonstrate in this research that the success of U-Net in the field of healthcare image classification is primarily attributed to the partitioning solution it employs, rather than the merging of different features. Half-U-Net is introduced as a result of this observation because it primarily facilitates the joint optimization part of the process. Unifying the channel numbers, employing feature-length fusion, and making use of Ghost modules are the three primary methods by which Half-U-Net reduces the complexity of the network. According to experimental findings, the proposed Half-U-Net achieved superior segmentation performance and reduced the network's complexity when compared to other U-Nets and their different versions. Omran Salih et al. [17] developed this article by using a binarization convolution operation instead of a standard convolution layer, creating a local binarization convolutional neural network (LBCDN) for comprehensive skin lesion segmentation, which greatly improved accuracy. The LBCDN architecture was proposed to minimize computational cost by combining an improved deep neural network with a smaller encoder and decoder system. The LBCDN framework achieved the highest dice coefficient among all other methods, demonstrating its superior and stable performance. Yadi Zhen et al. [18] highlight the challenges of defining the perimeter of melanoma due to its irregular shape, structure, and colour. In this paper, a better DC-U-Net network-based segmentation algorithm is developed to address these issues. To sharpen the model's focus on the melanoma lesion area, a connection-focused ECA-NET module has been added. In order to further refine the segmentation results, conditional random field and test data augmentation are used as post-processing techniques.

Zahraa E. Diame et al. [19] evaluated five frameworks (U-Net, Res-U-Net, VGG-16UNET, DenseNet-121, and EfficientNet-B0) to assess the potential application of deep learning techniques for skin lesion segmentation to identify lesion boundaries. The DenseNet-121 framework outperformed other methods in terms of precision rate across all training datasets. Prashant Brahmbhatt et al. [20] focus on segmenting the well-known skin lesion problem using an ensemble method. The paper combines the conventional strategy and the ensemble principle to achieve acceptable performance. The secondary goal is to reduce the time spent on image pre- and post-processing. The PH2 dataset consists of dermoscopic images of skin conditions and their corresponding ground truths, obtained through the traditional manual method.

Yangling Ma et al. [21] recommend a multi-instance, learning-based, end-to-end approach for melanoma identification and lesion segmentation simultaneously. They utilize multi-instance content based on a graph-convolutional neural network to recognize melanoma and fully leverage the information in high-resolution photos. The end-to-end approach treats segmentation and identification as inherently connected methods, where a high Jaccard index also indicates the stability of melanoma identification. Nawaz M et al. [7] present a deep learning approach to overcome the limitations of previous work. After completing the pre-processing procedure, they utilize the Corner-Net framework, an image detection technique, to diagnose melanoma lesions. The regional moles are then subjected to the fuzzy K-means clustering method for semantic segmentation. The proposed strategy is evaluated using two standard databases, ISIC-17 and ISIC-18, to assess its segmentation ability. Multiple tests have been conducted to demonstrate the reliability of the proposed strategy, using both numerical metrics and visual representations. Baiju Babu Vimala et al. (2023) [22] employ a hybrid deep learning approach to clean up breast ultrasound images with local speckle noise. Initially, logarithm and exponential modifications are applied to improve the brightness of ultrasonography breast images, followed by the use of guided filter techniques to enhance the detail of proliferative ultrasonography images.

Saravanan et al. [23] sparse coding estimates is utilised for higher-dimensional data, and the encoding scheme employed is metadata-based vector encoding. The atoms of nearby limitation are constructed on the basis of a well-organized k-neighboured system, which preserves the geometric structure of the supervised data. Saravanan et al. [24] kirsch's edge detectors detect boundary edge pixels, which are contrast adaptive histogram equalised. This augmented brain image is then Ridgelet transformed to obtain Ridgelet texture feature parameters. Features are obtained from Ridgelet transformed coefficients, enhanced using Principal Component Analysis, and categorized into Glioma or non-Glioma brain pictures using Co-Active Adaptive Neuro Fuzzy Expert System classification. Shivangi et al. [25] an exhaustive empirical assessment of convolutional neural networks (CNNs) applied to large-scale image classification of gait signals converted into spectrogram images and deep dense artificial neural networks (ANNs) employed for voice recordings has showcased remarkable superiority over existing state-of-the-art methods in disease prediction. The VGFR Spectrogram Detector achieved an impressive classification accuracy of 88.1%, while the Voice Impairment Classifier attained a remarkable 89.15% accuracy. Saravanan et al. [26] leveraging the automated feature extraction capabilities of a three-dimensional deep convolutional autoencoder (3D-DCAE), a novel method has been developed that integrates a neural network-based classifier to construct a unified framework capable of supervised training, achieving the pinnacle of classification accuracy for both ictal and interictal brain state signals. To thoroughly evaluate our method, two distinct models were meticulously crafted and assessed, employing three separate EEG data section lengths and a rigorous tenfold cross-validation procedure.

## U-shaped encoder-decoder network architecture

**Fig. 1:**
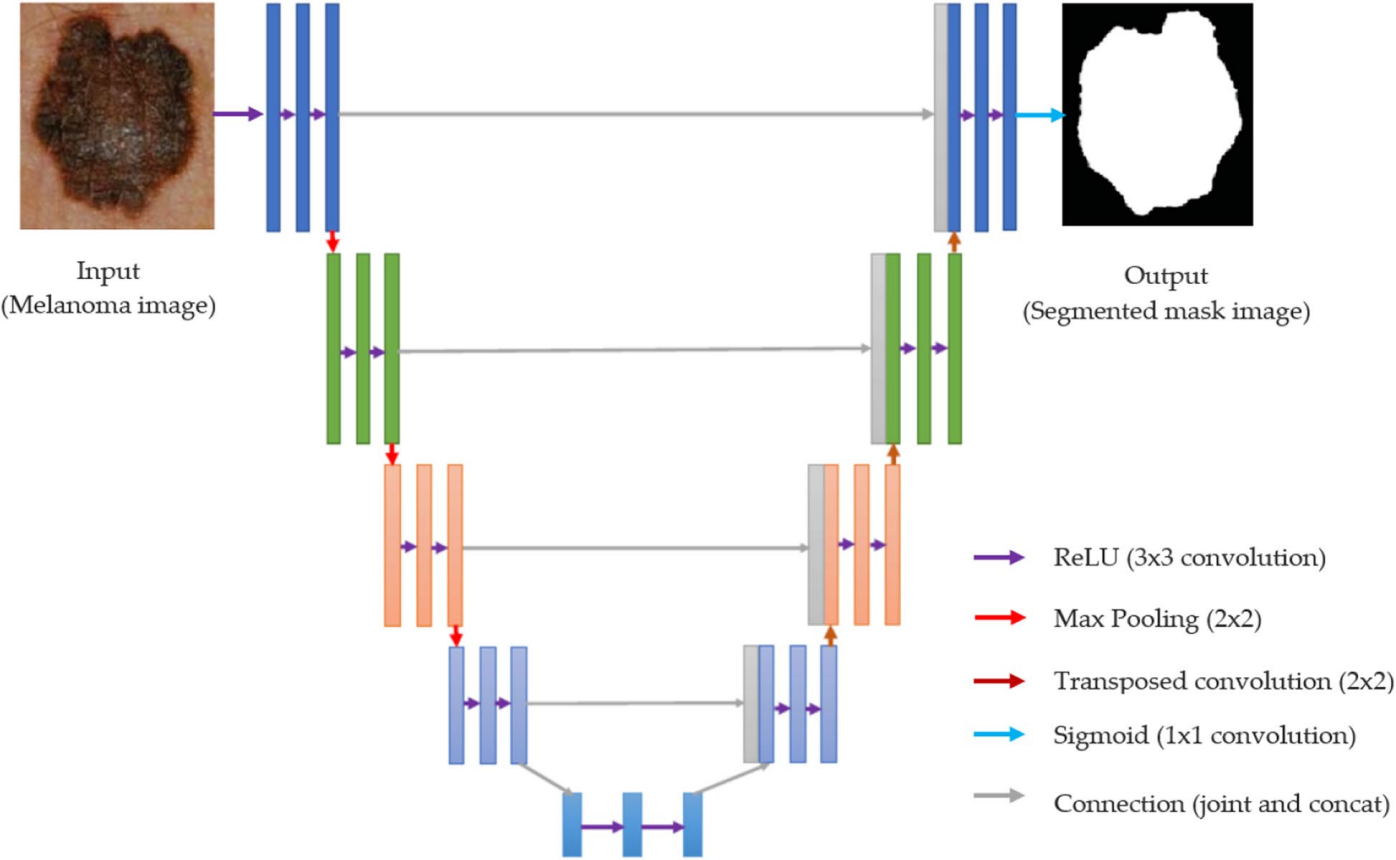
Architecture of U-shaped encoder-decoder network

In order to accomplish semantic segmentation, the U-Net employs a fully convolutional network, which is similar to the semantic segmentation method and a fully convolutional network method. The network is constructed to be symmetrical, with an encoder that detects spatial features in the image, and a decoder that uses those features to build a segmentation map. The encoder utilizes a conventional convolutional network architecture. It begins with a pair of 3×3 convolutions, followed by a max pooling function with a pooling dimension of 2×2 and a stride of 2. This process is repeated four times, doubling the number of convolution layer filters after each downsampling. The encoder is connected to the decoder through a series of two 3×3 convolutional operations. On the other hand, the decoder first up-samples the feature map using a 2×2 transposed convolutional operation, resulting in a reduction in the number of feature channels by half. This is followed by a sequence of two 3×3 convolutional operations. This upsampling and two-convolutional processing cycle is repeated four times, with the number of filters in each cycle halved to match the encoder. The final segmentation map is created using a 1×1 convolutional technique. The Rectified Linear Unit (ReLU) activation function is used by all layers except the last convolutional layer, where the sigmoid activation function is employed. One of the most distinctive features of the U-Net design is the use of skip connections. The output of the convolutional layer is passed on to the corresponding layer in the decoder before the pooling process of the encoder. Figure 1 depicts the architecture of U-Net, incorporating an encoding path and a decoding path with skip connections between the appropriate layers.

The up-sampling procedure results in concatenated feature maps, which are then propagated to the subsequent layers. This allows the network to recover spatial features that may have been lost during pooling operations, thanks to the skip connections. In the context of parametric segmentation, the U-Net design was modified to become a three-dimensional U-Net. This involved replacing the 2D convolution max-pooling and asymmetrical operations with their 3D counterparts. To reduce the number of variables, however, the depth of the network was reduced by one, and the number of filters was doubled before the pooling layers to avoid bottlenecks. In the original U-Net, batch normalization was not used. However, batch normalization was tested in the 3D U-Net, and surprisingly, the results indicated that batch normalization could sometimes decrease performance.

### Modification of scale factor - medical images

**Fig. 2:**
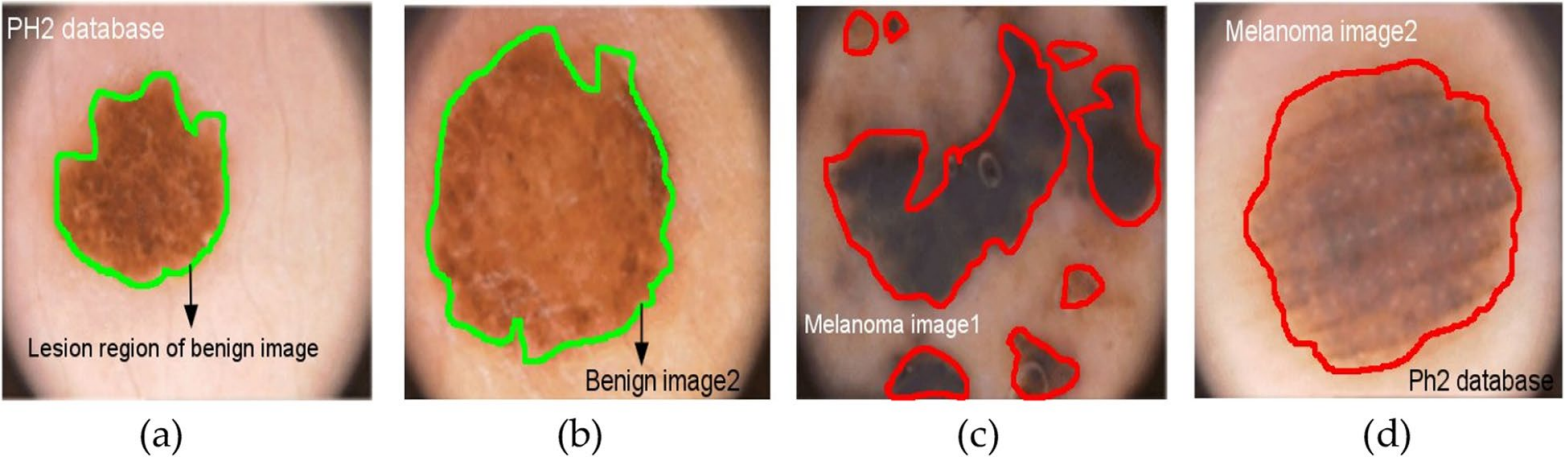
Sample lesion-dermoscopic images from PH^2^ database; **a**, **b** sample images of benign type; **c**, **d **sample images of melanoma type

In medical image analysis, our goal is to separate nuclei, organ systems, and carcinomas from image data captured by different types of equipment. However, these objects of interest often exhibit non-uniform and varying scales. For example, as shown in Fig. 2, dermoscopy images can vary greatly in the size of skin lesions. These sample lesion dermoscopy images are obtained from the PH2 database [27]. Such variations are commonly encountered in various medical image segmentation tasks. Therefore, a network's ability to handle such variations becomes crucial in performing analyses on different entities at different levels. While several deep vision-based studies have addressed this issue, it has not been adequately addressed in the field of medical semantic segmentation. The U-Net framework utilizes a series of two 3×3 convolution operations after every pooling layer and transposed convolution layers. This two-step convolution process is similar to a 5×5 convolution procedure. To enhance U-Net with multi-dimensional analysis, an effective approach is to incorporate 3×3 and 7×7 convolutional processes alongside the 5×5 convolution layer. By replacing the convolution layers with blocks similar to Inception, the U-Net architecture can better balance the learned characteristics from the image at different sizes. Although depth-wise convolutions could be considered, our testing phase showed that incorporating Inception-like blocks outperformed this approach. Despite the performance improvement, it's important to note that including more convolution layers in parallel may increase the memory requirements.

**Fig. 3:**
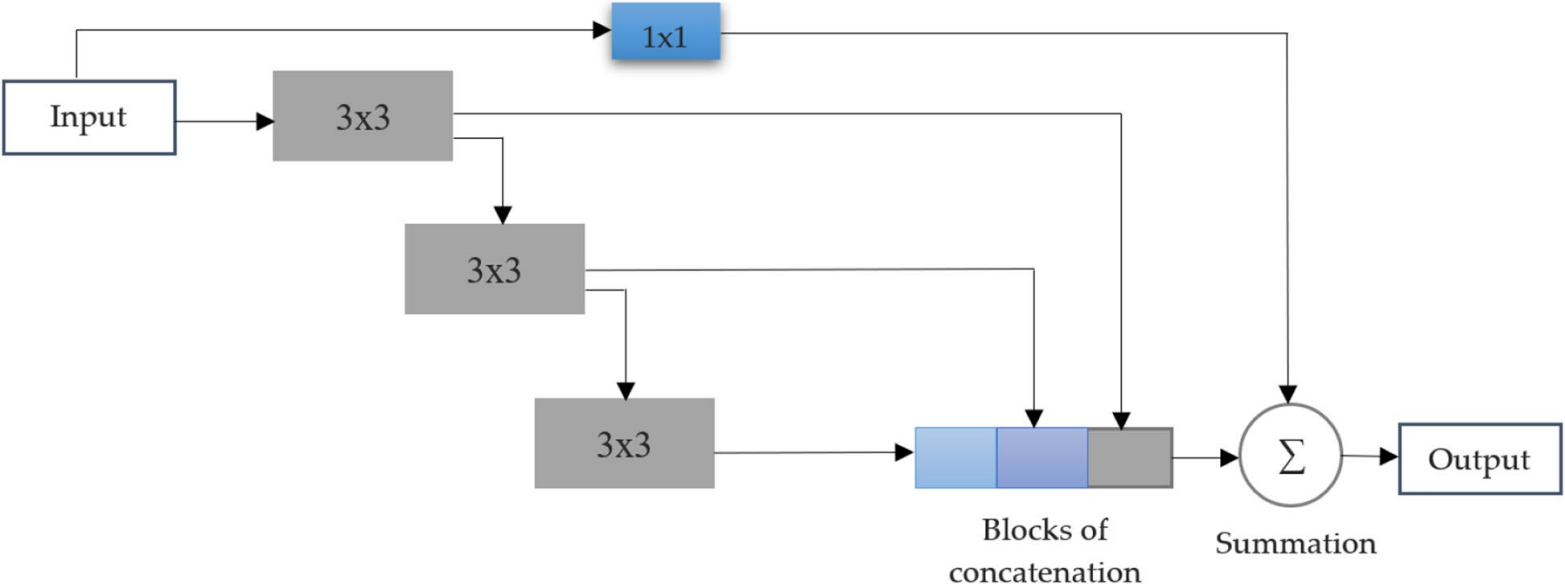
Multi-dimensional block

**Fig. 4:**
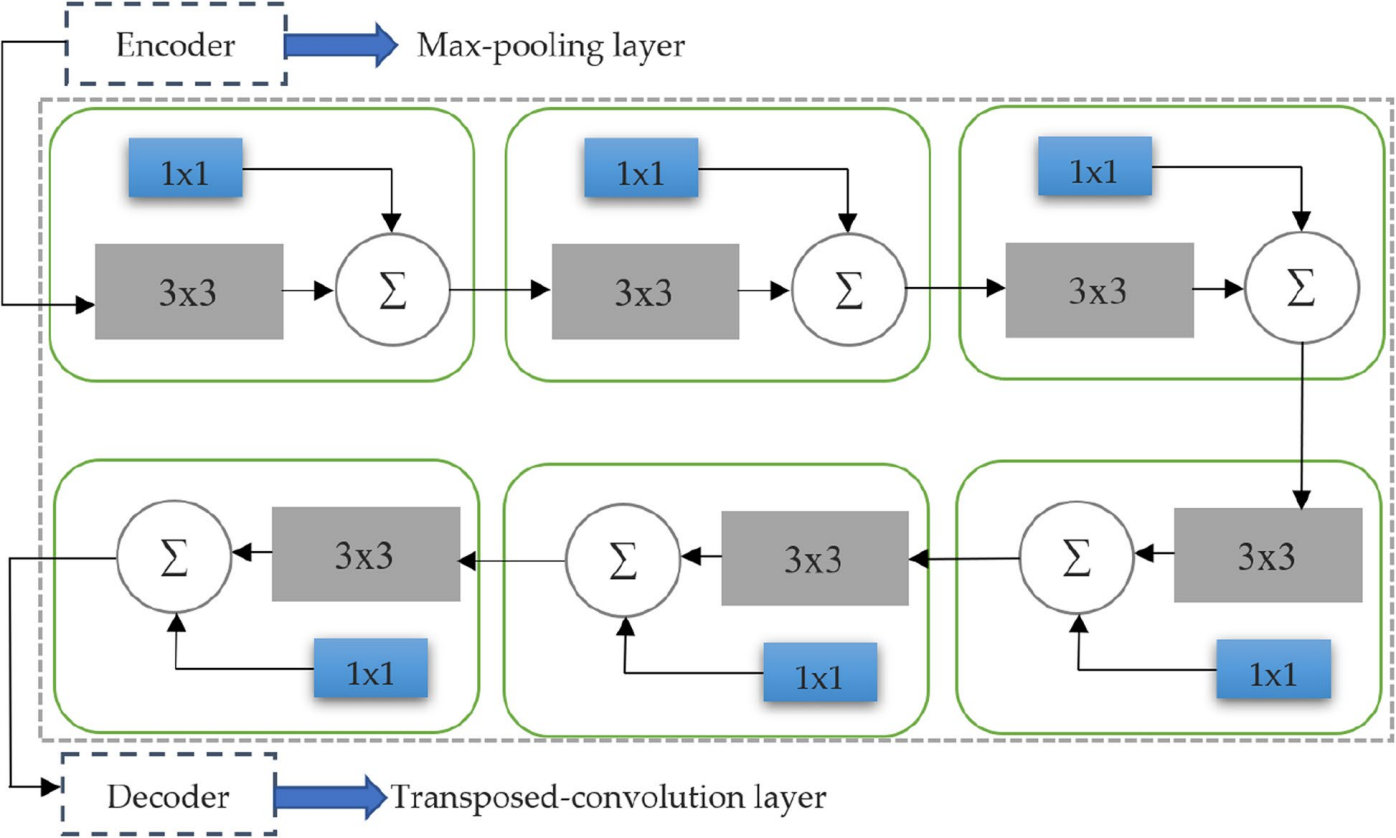
Proposed encoder-decoder convolution path

We utilize a series of compact 3v3 convolution blocks to factorize the larger and more challenging 5×5 and 7×7 convolutional layers. The second and third 3×3 convolution blocks yield results that closely resemble those of 5×5 and 7×7 convolutions, respectively. To capture spatial characteristics at various scales, we collect the outputs from these three convolution blocks and concatenate them together. Our research findings indicate that the results from this condensed block are nearly identical to those from the memory-intensive Inception-like structure. This supports the hypothesis that adjacent layers in a visual network are correlated, and this modification significantly reduces the required memory. The memory impact is primarily due to the quadratic increase in the number of filters in the first convolutional layer in deeper network models. To avoid excessive memory utilization, instead of maintaining an equal number of filters in each of the three successive convolutional layers, we gradually increase the number of filters in specific layers (from one to three) to prevent the memory load from the previous layer from propagating excessively into the core part of the network. This allows us to capture spatial attributes derived from distinct context sizes. However, we do not use 3×3, 5×5, and 7×7 filters simultaneously; rather, we interpolate the larger and more expensive 5x5 and 7×7 filters as a series of 3×3 filters (as shown in Fig. 4). Additionally, we incorporate a residual network, known for its effective segmentation process in biomedical images, and the inclusion of 1×1 convolution layers, which provide additional spatial information interpretation. Both of these factors contribute to the success of our approach (as shown in Fig. 3). The multi-dimensional block demonstrates how we progressively increase the number of filters in the three layers following it, along with the inclusion of a residual network (and a 1×1 filter to maintain dimensions).

### Possible conceptual difference between encoder-decoder levels

An intensive U-Net model contribution was the prime reason for making shortcut links between the respective layers, and it is placed prior and after the max-pooling, de-convolutional layers, respectively. It lets the network send from encoder to decoder the spatial details that may have been lost during the pooling process. Maintaining dispersed spatial features, a shortcut link establishes a connection between the encoder that comes prior to the first pooling and the decoder that comes after the final deconvolution process.

When considering the assumptions regarding encoder feature inputs in neural networks, it has been observed that these inputs originate from lower-level computations in the network. Conversely, the features in the decoders are computed in deeper levels, resulting in higher-quality representations. However, performing this operation effectively may require a significant amount of available processing resources. Nevertheless, it is recognized that there may be a conceptual discrepancy when combining these distinct feature sets, which could potentially affect our prediction method during the learning stage. However, as we progress through the shortcut connections, the level of difference is expected to diminish considerably. This can be attributed to the integration of encoder and decoder functions, working together to achieve intensive processing. To address the feature gap between the encoder and decoder, we suggest integrating additional convolutional layers accompanied by shortcut links. This helps to reduce the disparities and compensate for any additional processing performed during decoding through nonlinear transformations on the propagated features. Additionally, we enhance the standard convolutional layers by incorporating residual connections, which have demonstrated significant capability in representing medical images. This concept draws inspiration from image-to-image conversion tasks performed using convolutional neural networks, where the use of pooling layers often results in data loss. Instead of simply appending the feature maps from the encoder to the decoder, we employ a series of convolutional layers with residual blocks, concatenating them with the features from the decoder stage. In Fig. 4, we demonstrate that the combination of encoder and decoder feature maps involves running the encoder features through several convolutional layers first. This is expected to narrow the conceptual differences in feature maps between the encoder and decoder through the application of nonlinear procedures. Additionally, the implementation of residual connections facilitates faster learning and is highly beneficial in deep neural networks.

## Proposed MultiDimensional U-CNN architecture

MultiDimensional U-CNN is a promising technology with the potential to revolutionize various medical fields. Its ability to accurately segment complex anatomical structures and pathological lesions can empower clinicians to make better-informed decisions, ultimately leading to improved patient outcomes. We replace the sequence of two convolutional layers in the MultiDimensionalU-CNN model with the proposed MultiDimensional block, which was discussed in “Proposed MultiDimensional U-CNN Architecture” section. We assign a parameter called *M* to each of the multidimensional blocks. This parameter governs the number of filters that are used in the convolutional layers that are contained within that block. We calculate *M* to ensure that the number of parameters in the original U-Net and those in the proposed model have a similar relationship.

1 $$M=\beta \times {F}_{n}$$

β—> scaler co-efficient, ${F}_{n}$—> number of U-Net layer filters.

**Fig. 5:**
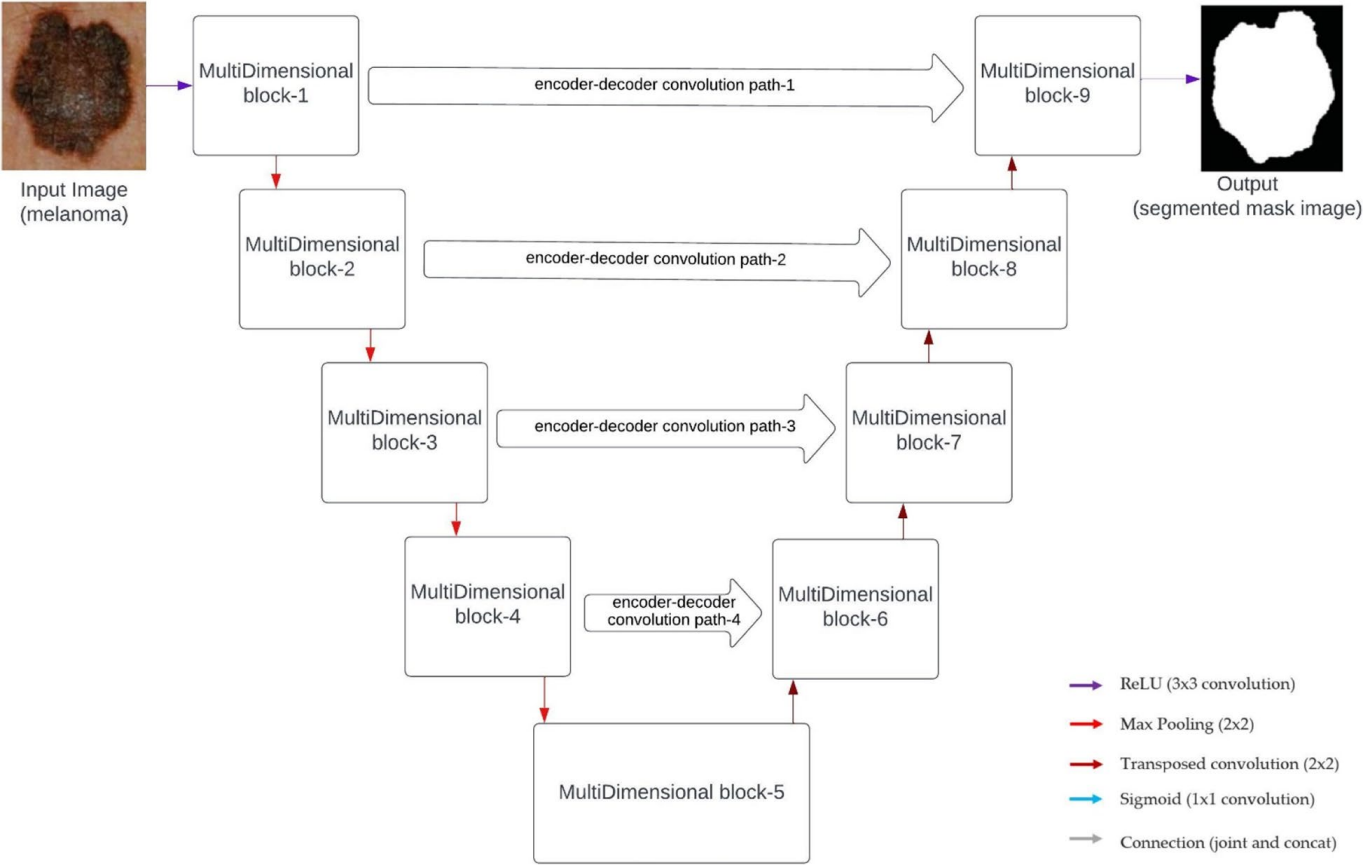
Proposed MultiDimensionalU-CNN architecture

Decreasing *M* into *F*_*n*_ and *β* is a practical way to cut down on the number of parameters while keeping them the same as in U-Net. Our recommended model is compared to a U-Net with filter sizes of [16; 32; 64; 128; 256; and 512] along the stages, and these values are similar to our *F*_*n*_. Since we wanted to keep the number of model parameters lower than the U-Net, we opted *β* = 1.56. We emphasised the importance of gradually increasing the number of filters in the subsequent convolutional layers within a multidimensional block rather than maintaining the same number. Assigning filters $\left[\frac{M}{6}\right],\left[\frac{M}{3}\right]$ and $\left[\frac{M}{2}\right]$ to the three convolutional layers in order yielded the best results in our research. Also, *M* is doubled after every pooling and deconvolution operation, just like the U-Net. In addition to the MultiDimensional blocks, the proposed encoder-decoder convolution path would replace the normal shortcut connections. For this reason, we perform a few convolutional processes on the extracted features that are being sent from the encoder to the decoder. By getting closer to the internal shortcuts, we expect the semantic difference between the decoder and encoder feature maps to be less noticeable. All along the encoder-decoder convolutional path, we also employ a decreasing number of convolutional blocks. The Rectified Linear Unit (ReLU) activation function is utilized in this network for all of the convolutional layer activation, with the exception of the output layer. All the convolutional layer activation in this network is batch normalized. The output layer, such as the U-Net model, is activated by a sigmoid activation function. Figure 5, depicts the architecture of proposed MultiDimensionalU-CNN architecture. The proposed multidimensional block replaces the sequences of two convolution layers in U-Net frameworks. In addition, rather than using simple shortcut connections, we employ the proposed encoder-decoder convolution paths. Table 1 represents the detailed description of MDU-CNN architecture.

**Table 1:** Detailed architecture of MultiDimensionalU-CNN

MultiDimensionalU-CNN					
**Block Name (B)**	**Size of the Filter**	**No. of filters**	**Convolution Path**	**Size of the Filter**	**No. of filters**
MultiDimensional B1	2D-conv(3,3)	7	encoder-decoder convolution path-1	2D-conv(3,3)	16
	2D-conv(3,3)	18		2D-conv(3,3)	16
MultiDimensional B9	2D-conv(3,3)	26		2D-conv(3,3)	32
	2D-conv(1,1)	52		2D-conv(1,1)	32
MultiDimensional B2	2D-conv(3,3)	18		2D-conv(3,3)	32
	2D-conv(3,3)	36		2D-conv(3,3)	32
MultiDimensional B8	2D-conv(3,3)	54		2D-conv(3,3)	32
	2D-conv(1,1)	106		2D-conv(1,1)	32
MultiDimensional B3	2D-conv(3,3)	36	encoder-decoder convolution path-2	2D-conv(3,3)	64
	2D-conv(3,3)	73		2D-conv(3,3)	64
MultiDimensional B7	2D-conv(3,3)	107		2D-conv(3,3)	64
	2D-conv(1,1)	214		2D-conv(1,1)	64
MultiDimensional B4	2D-conv(3,3)	72		2D-conv(3,3)	64
	2D-conv(3,3)	144		2D-conv(3,3)	64
MultiDimensional B6	2D-conv(3,3)	216	encoder-decoder convolution path-3	2D-conv(3,3)	128
	2D-conv(1,1)	428		2D-conv(1,1)	128
MultiDimensional B5	2D-conv(3,3)	146		2D-conv(3,3)	128
	2D-conv(3,3)	292		2D-conv(3,3)	128
	2D-conv(3,3)	428	encoder-decoder convolution path-4	2D-conv(3,3)	256
	2D-conv(1,1)	856		2D-conv(1,1)	256

### Experimental setup-dataset

Medical imaging dataset curation is more difficult than conventional computer-aided dataset curation. The high cost of imaging equipment, the complexity of image acquisition pipelines, the need for expert interpretation, and concerns about privacy make it hard to make medical imaging datasets. To this end, only a limited number of publicly available benchmark datasets for medical imaging exist, each of which contains only a few examples of diagnostic images. We wanted to examine the performance of proposed framework by putting it through a number of different imaging techniques. In particular, we chose datasets that were as different as possible from one another. Table 2 contains the detailed description of various datasets utilized in the proposed system.

**Table 2:** Detailed description of various dataset

Methods	No. of dataset	Total number of images	Image resolution	Required resolution (input)
Magnetic resonance image	BraTS2020	230 high-grade, and 85 low-grade gliomas	240 × 240 × 155	80 × 80 × 48
Non-invasive dermoscopy	ISIC2020	3213	not consistent	256 × 192
Microbes Fluorescence Microscopy	Murphy Lab 3D hela	106	not consistent	256 × 256
Endoscopy	EndoSLAM	714	384 × 288	256 × 192
Electron Microscopy	ISBI-2022	45	512 × 512	256 × 256

#### Magnetic resonance image

All kinds of previously mentioned datasets include 2D clinical images, then we used MRI images from the BraTS2020 database in order to examine the feasibility of our proposed framework for 3D medical imaging. This dataset contains 210 glioblastoma (HGG) and 75 lower-grade glioma (LGG) multimodal MRI scans [28]. These multimodal scans include native (T1), post-contrast T1-weighted (T1Gd), T2-weighted (T2) and T2 Fluid Attenuated Inversion Recovery (FLAIR) volumes, which were acquired following different clinical protocols and various scanners. The images are of dimensions 240 × 240 × 155 but have been resized to 80 × 80 × 48 for computational ease. All the four modalities, namely, T1, T1Gd, T2 and FLAIR are used as four different channels in evaluating the 3D variant of our model.

#### Non-invasive dermoscopy

The images of dermoscopy were obtained from the ISIC2020 database (Lesion Boundary Segmentation Task). The ISIC2019 dataset and the HAM-10000 dataset were used to get the data for this task [29]. There are a total of 3213 images of various skin lesions, all of which have expert annotations. The images were originally at a wide variety of resolutions but were scaled down to 256×256 while preserving their median aspect ratio for fast computation.

#### Microbes fluorescence microscopy

The Murphy Lab's dataset of microbe’s fluorescence microscopic analysis was used for this research. The 106 fluorescent microscope image dataset contains a maximum of 4106 cells. There are 50 percent U2OS cells and 50 percent NIH3T3 cells, and the nuclei were manually segmented by specialists [30]. This dataset of the bright-field microscopy images is difficult to analyse because of the inconsistent brightness of the nuclei and the presence of visible debris. Due to computational constraints, the actual size of the image’s ranges from 1349×1030 - 1344×1024, but they have been scaled to 256×256.

#### Endoscopy

The EndoSLAM, a gastrointestinal image database, was utilised for our endoscopy image investigations, and this dataset contains images extracted from 38 video sequences of colonoscopy. However, the images that contained polyps were taken into consideration, resulting in the collection of 714 images [31]. The images had a resolution of 386×292 when they were first captured, after it has been downsized to 256×256 while keeping their aspect ratio.

#### Electron microscopy

We used the dataset from the ISBI2022: 2-dimensional EM segmentation task to analyse the efficacy of the framework with electron microscope images. A total of 45 images from ssTEM (transmission electron-microscopy) of the ventral nerve cord of Drosophila first instar larvae are included in this collection [32]. The images originally had a resolution of 640×480 pixels, but because of computational constraints, they were downsized to 256×256 pixels.

## Experimental analysis and baseline model

**Fig. 6:**
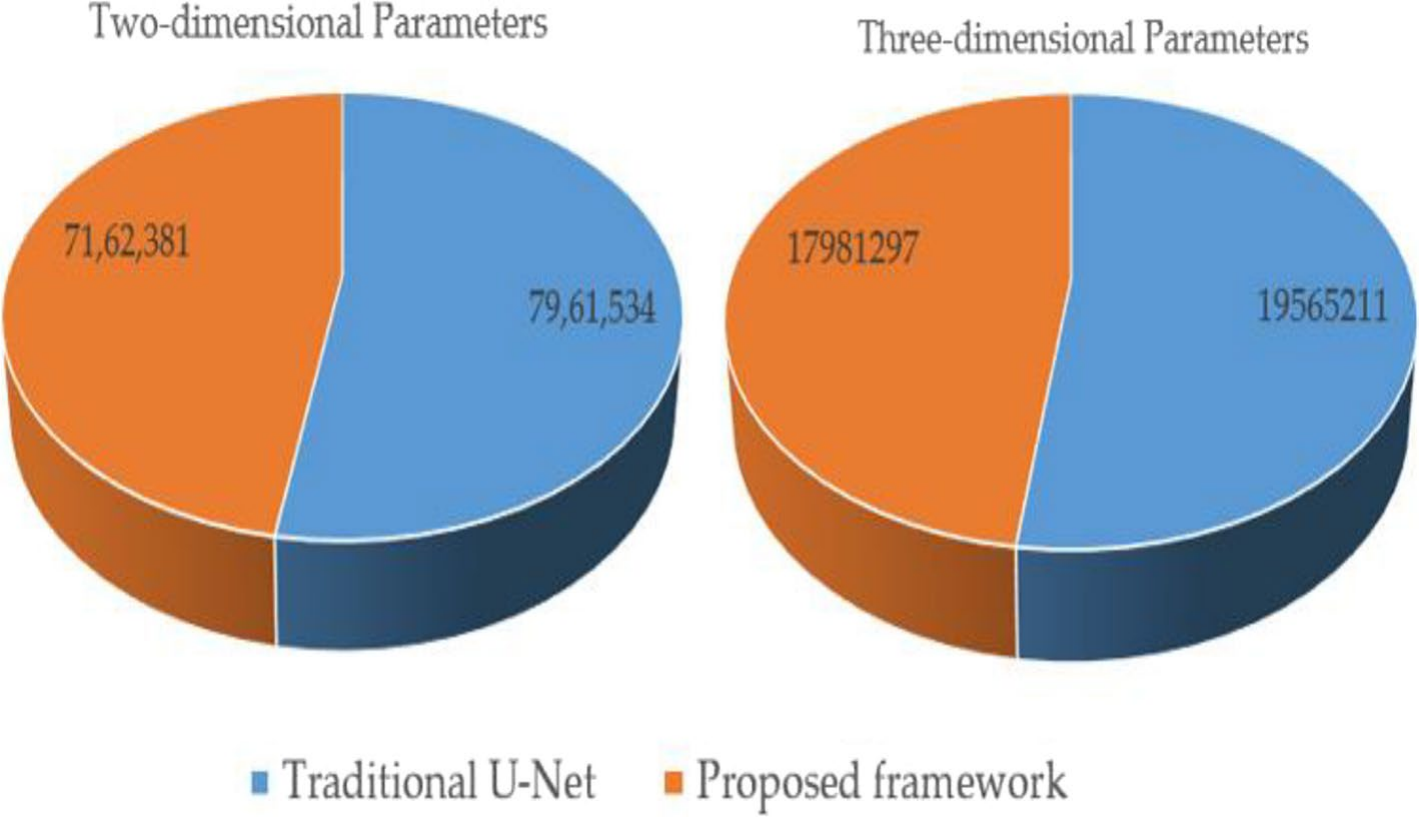
Proposed and traditional framework parameter utilization demonstration

Python's programming language known as Pyhton3 was utilized in conducting our study. By utilizing Keras and its Tensor Flow backend we were able to construct the network models. To execute the tests have been used on a laptop computer that is integrated with features such as Intel Core i5-7700 Processor having frequency up to 4.4 GHz and also comprised with enough Memory space up to 16 GB RAM along-with-Graphics which is powered by MSI Nvidia RTX 3060 having enough strength which is around 12 GB GDDR6, to identify the most effective segmentation approach for clinical images we compared how well MultiDimensionalU-CNN performed relative to U-net. To maintain consistency in the number of parameters between our proposed MultiDimensionalU-CNN model and Classic U-net model we employed a six-layered deep encoder-decoder configuration on latter ensuring filters range from as low as 16 through till high-end i.e., 512. Building a 3D version of MDU-CNN can be easily done by just replacing all its core components with their appropriate three-dimensional counterparts, and there aren't any extra changes or additions that take place during this phase of construction. The different dimensional methods and its respective parameter utilization by the proposed and traditional U-Net model information is shown in Table 3 and Fig. 6.

**Table 3:** Different dimensional method and parameters used in proposed method

Two-dimensional		Three-dimensional	
**Type**	**Parameters**	**Type**	**Parameters**
Traditional U-Net	7,961,534	Traditional U-Net	19,565,211
Proposed framework	7,162,381	Proposed framework	17,981,297

### Image resizing and training mode

The goal of the experiment analysis is to examine the proposed MDU-CNN architecture is better than the original U-Net model. As a result, there was no pre-processing done that was specific to the domain. Input images were just downscaled so they would fit in the GPU-memory, and their image pixels were partitioned by 255 so they would fall in the [0:::1] range before being processed. Similarly, there was no post-processing done that was specific to the domain as well. Because the activation function of the last layer is a Sigmoid, its output values are also in the range [0:::1]. Hence, we used a 0.5 threshold to produce the input image segmentation map. The goal of semantic segmentation is to determine, for each individual pixel, whether it is a potential point of interest or simply a part of the background. As a result, we may simplify this issue to a binary classification problem on a pixel-by-pixel basis. As a result, in order to calculate the loss function for the network, all we did was minimize the binary cross-entropy factor. Let, *A* is an image, and *B* is a segmentation mask of ground truth, it can be computed by the given model is $\widehat{B}$. Here, for pixel ${p}_{i}a$, then the network computes ${\widehat{b}}_{{p}_{i}a}$, likewise the ${b}_{{p}_{i}a}$ is the value of the ground truth. The definition of that image's binary cross-entropy loss is as follows:

2 $$Cros\;Entropy\;\left(A,B,\widehat B\right)=\sum\nolimits_{p_ia\in A}-(b_{p_ia}\text{log}\left({\widehat b}_{p_ia}\right)+\left(1-b_{p_ia}\right)\text{log}(1-{\widehat b}_{p_ia}))$$

When, the batch is having ‘*m*’ images, then the function of loss (*I*) can be written as,

3 $$I=\frac1m\;{\textstyle\sum_{i=1}^m}Cross\;Entropy\left(A_i,B_i,\widehat{B_i}\right)$$

We tried to reduce the loss of binary cross-entropy as much as possible, hence we trained the model via stochastic gradient descent optimizer. Estimates of the initial and second values of the gradients are used in stochastic gradient computation of diverse learning levels appropriate for each of the parameters. The stochastic gradient is equipped with a set of parameters, two of which, *α1* and *α2*, are responsible for controlling the rate of decay of the first and second moments, respectively. To train the models, a stochastic gradient optimizer was used for a total of 150 iterations. The number of iterations was set at 150 since, after that point, there was no apparent progress in either model.

### Performance metric evaluation

During the semantic segmentation process, the places of interest usually make up a small part of the whole image. Metrics like precision and recall are insufficient and frequently lead to a misleading perception of superiority, which is augmented by the faultless detection of the background. As a result, the Jaccard Index has found widespread application in the process of evaluating and benchmarking various segmentation methods and object localization techniques. The Jaccard index (*JI*) is defined with two pairs, *X* and *Y*, as the proportion of the set’s intersection and association. This ratio can also be written as,

4 $$JI=\frac{intersection}{association}=\frac{X\cap Y}{X\cup Y}$$

In this particular scenario, set *X* denotes the binary-segmentation mask *B* that correlates to the ground truth, and set *Y* relates to the binary segmentation mask $\widehat{B}$ that was predicted. As a result of using the *JI* as the measure, it is not only emphasising accurate segmentation, but it also penalises both under and over segmentation.

### K-fold cross validation

Leave-one-out cross-validation methods are used to test an algorithm's overall performance on a dataset that is not dependent on it. These tests keep a proper balance between variability and bias. The dataset *C* is subclassified in to k mutually unique subsets ${C}_{1},{C}_{2},{C}_{3}, \dots .{C}_{k}$ for the k-fold cross-validation test. Each iteration of the algorithm uses one of the *k* divides as the testing dataset and the remainder as the learning set. To examine the segmentation performance of the traditional U-Net and the proposed MultiDimensionalU-CNN framework, Leave-one-out cross-validation tests were carried out on each of the distinct datasets. However, it is a supervised neural pipeline, and the highest performing outcome on the testing set was attained over the maximum quantity of epochs (150) that were carried out in each instance. In conclusion, an average assessment of the effectiveness of the algorithm may be obtained by merging the outcomes of all *k* separate iterations.

## Results and discussions

Experiments were conducted using a wide variety of medical image types, each presenting its own specific set of challenges, to evaluate the effectiveness of the proposed framework. Specifically, we performed a Leave-one-out cross-validation and compared the effectiveness of our proposed MultiDimensionalU-CNN to that of the conventional U-Net. Throughout the 150 epochs carried out, the better outcomes achieved on the testing dataset were recorded during each iteration, and these records were aggregated to produce the final result. Table 4 displays the results of the cross-validation using the leave-one-out criterion, applied to each dataset using both the proposed MDU-CNN framework and the classic U-Net model. In this section, we present the most successful results obtained with U-Net and MDU-CNN across all five folds of the datasets. Furthermore, we discuss how MDU-CNN represents a significant improvement over U-Net in terms of quality. It is important to note that the decimal Jaccard indexes have been transformed into percentage values (%) to enhance the readability of the table. Table 4 demonstrates that the performance of our proposed framework surpasses that of the conventional U-Net architecture in the classification of various medical image types. Notably, images obtained through dermoscopy and endoscopy have shown remarkable improvements.

**Table 4:** Comparison outcomes of proposed and traditional method validation accuracy based on k-fold cross-validation

Type	Proposed MDU-CNN (%)	Traditional U-Net (%)	Comparative Increase in performance (%)
Magnetic Resonance Image	79.2130 ± 0.8182	77.2289 ± 0.6923	1.3241
Non-invasive dermoscopy	81.3188 ± 0.4423	76.1256 ± 3.9834	5.1932
Microbes Fluorescence Microscopy	92.6228 ± 0.9816	88.1209 ± 1.9923	4.5019
Endoscopy	83.1567 ± 1.6822	72.9190 ± 1.3989	10.2377
Electron Microscopy	88.8651 ± 0.8012	87.9929 ± 0.7717	0.8722

**Fig. 7:**
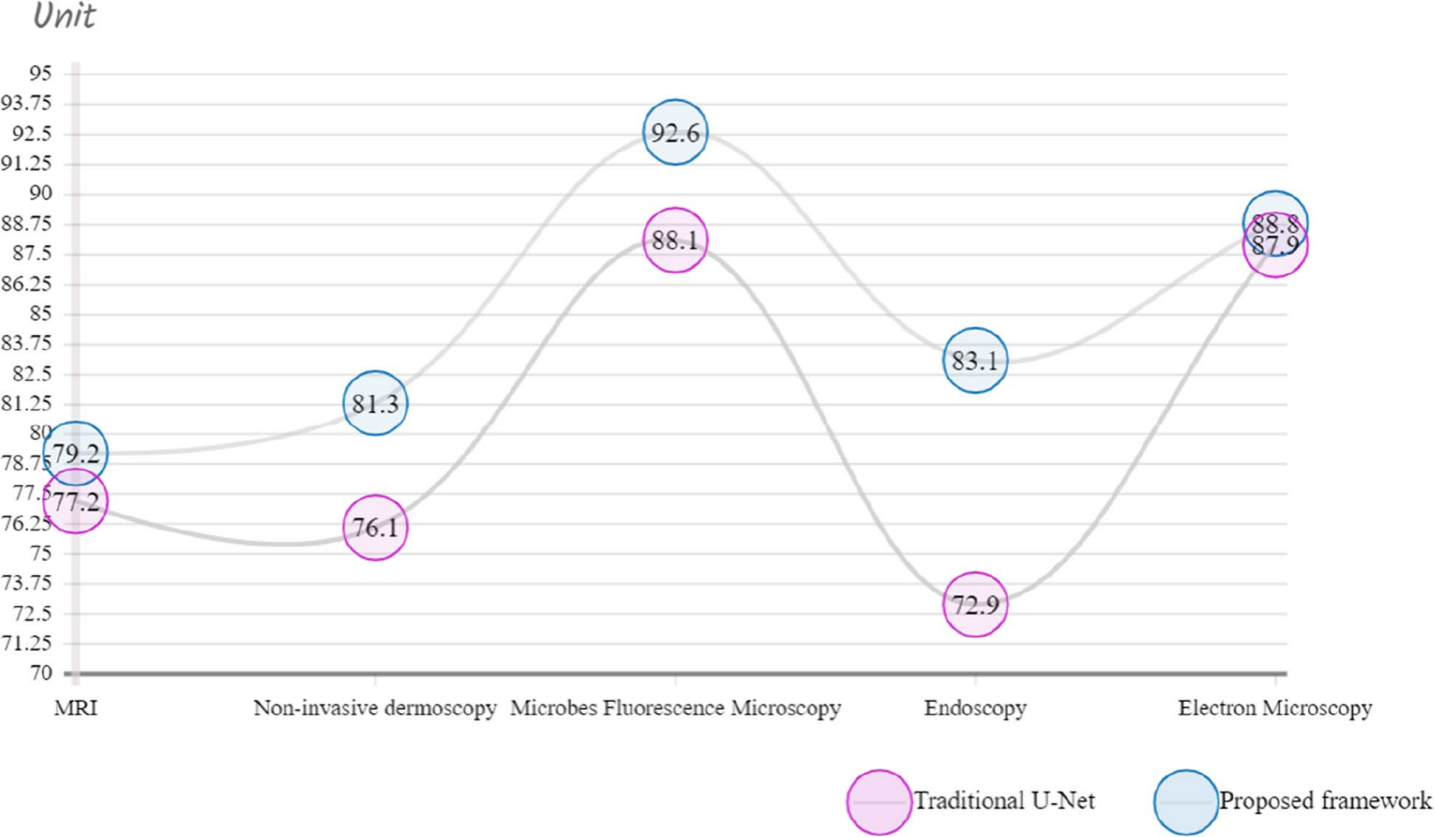
Graphical chart of comparison outcomes of traditional and proposed MDU-CNN validation accuracy by Leave-one-out cross-validation

**Fig. 8:**
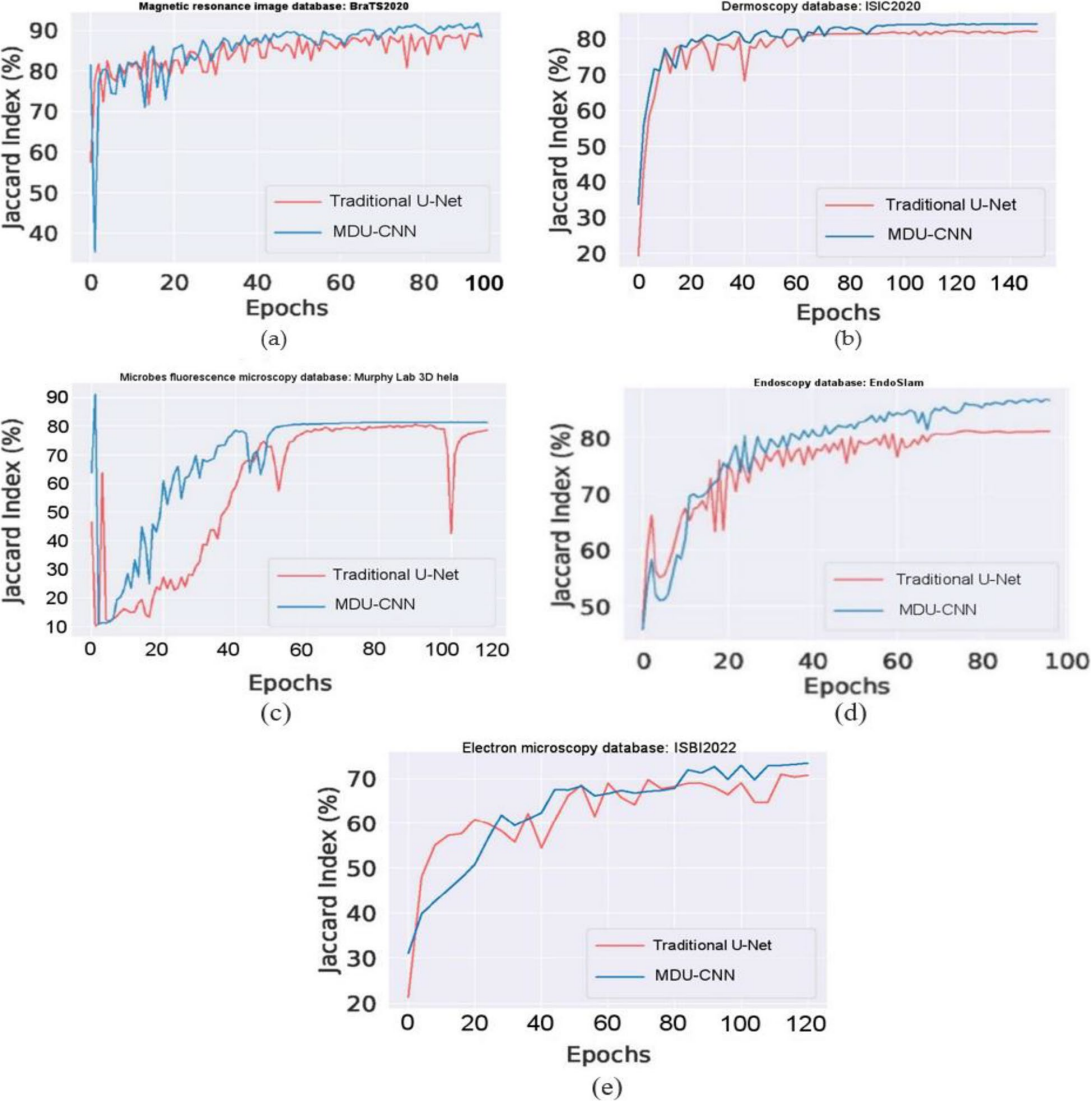
Improvement in testing efficiency as measured by epoch count **a** MRI; **b** dermoscopy; **c** fluorescence; **d** endoscopy and **e** electron microscopy

Despite its lower parameter count compared to that of U-net our model achieves great results when it comes to image analysis; specifically, a relative improvement over U-net by 4.5019% for the analysis microscopic fluorescence images and an enhance performance of up to 1·3241% for magnetic resonance imaging (MRI). The depiction of outcomes using Leave-one-out Cross validation for traditional and MDU-CNN can be seen in Fig. 7. To determine which models performed best in every trial we analyzed their overall improvement across all epochs. Figure 8, the outcomes of evaluating all of the datasets based on their effectiveness on the testing dataset at each epoch are demonstrated. Within the context of the cross validation, we have shown the range of Jaccard Indexes at a specific epoch. All the different scenarios shows that the proposed framework gets closer to the right answer much faster. It could be explained by the complementary relationship that exists between batch normalization and residual connections. In addition to this, with the exception of Fig. 8e, the MDU-CNN model has actually outperformed the traditional U-Net model in all of the other instances. Figure 8e shows that the MDU-CNN model, which initially lags behind the traditional U-Net for the electron microscope images, then ultimately converges at a higher accuracy over U-Net. A further interesting result from the studies is that, with the exception of a few insignificant operations, the confidence interval for the efficiency of the MDU-CNN is significantly lower. This finding demonstrates the dependability and consistency of the developed framework. This suggests that the proposed MDU-CNN framework can get better results with less training time than the traditional U-Net design.

**Fig. 9:**
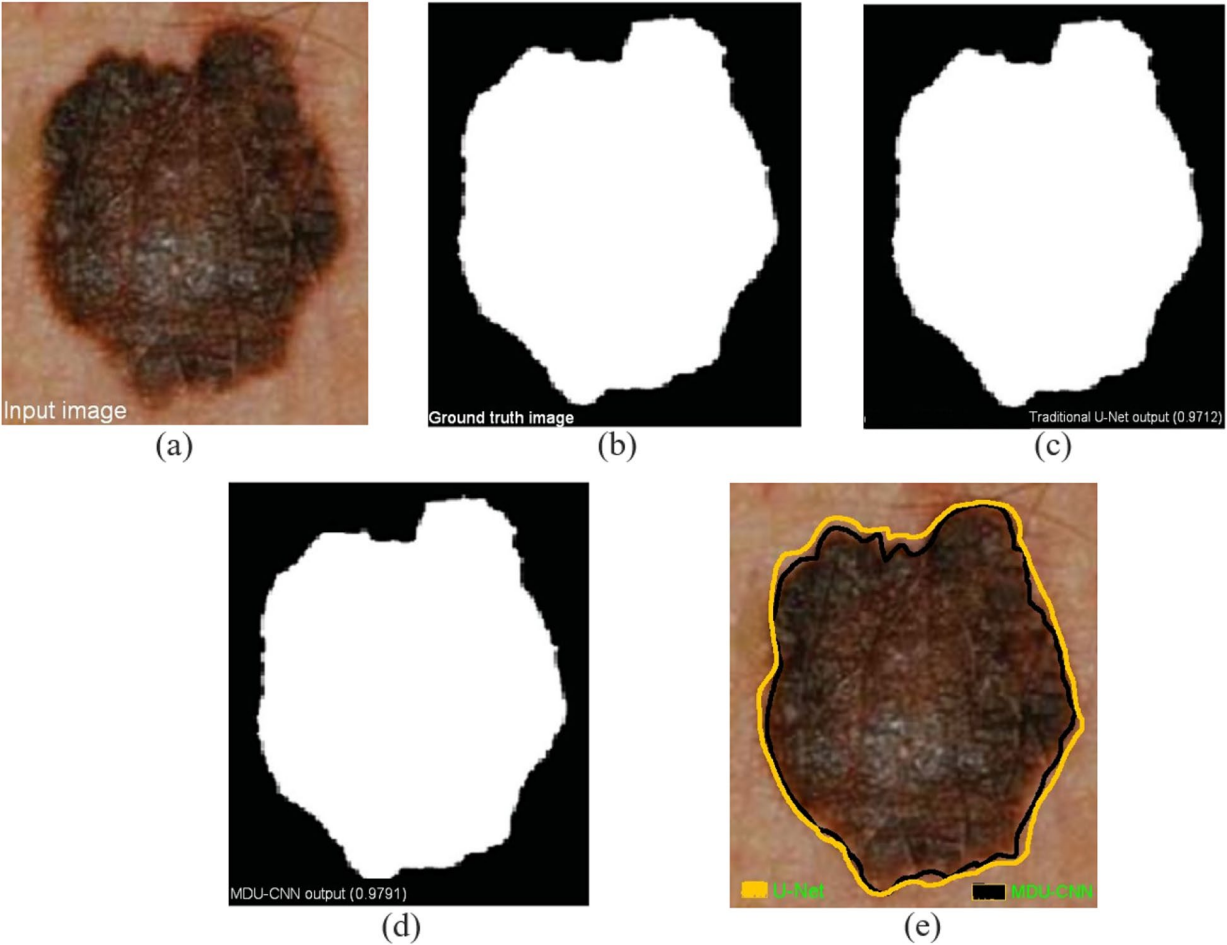
Polyp with distinct edge identified and segmented **a** input image; **b** ground truth; **c** traditional U-Net; **d** MDU-CNN and **e** final image

**Fig. 10:**
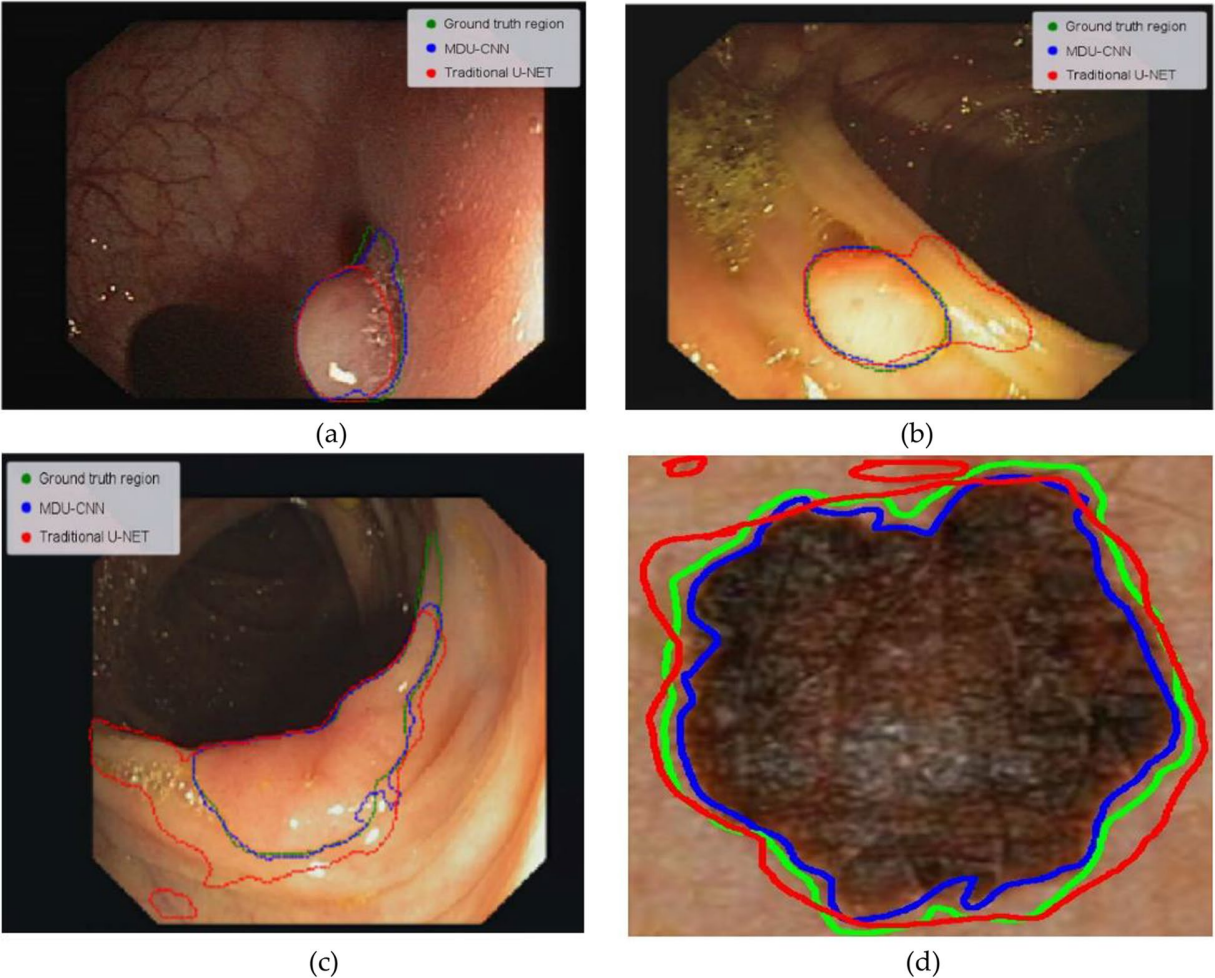
Boundary-less image segmentation

U-Net, which is the model that represents the state of the art in terms of medical image segmentation at the moment, has shown quite excellent outcomes in our studies. In Fig. 9, for instance, the U-Net model successfully segments a polyp with a significant Jaccard index due to its well-defined boundary; however, our proposed model performs marginally better. U-Net achieves impressive performance in polyp segmentation (Jaccard index = 0.9712). Relatively improved segmentation can be achieved with MDU-CNN (Jaccard index = 0.9791). U-Net appears to be having some trouble as we analyse more difficult images, especially ones with less apparent boundaries (Fig. 10). Lack of sharp resolution is a common problem in images of colon polyps. U-Net model under-segmented (Fig. 10a) or over-segmented (Fig. 10b) polyps in such circumstances. But in the other side, the proposed MDU-CNN that we proposed performed significantly more effective in both of the instances. Figure 10c displays the results of a comparison between the two models, showing that MDU-CNN performs better in the circumstances where both models encounter difficulties. Even though dermoscopy pictures often have more clearly defined areas, Fig. 10d shows that MDU-CNN is better at outlining borders. Similarly, it was seen with various different image types. We speculate that MDU-CNN can achieve more pixel-perfect segmentation due to its ability to use a variety of filter sizes. Table 5, illustrates the list of technical short form and its respective abbreviations used in this article.

**Table 5:** The individual contributions of MultiDimensional blocks and encoder-decoder routes are being studied using ablation. Using 5-fold cross-validation, the EndoSLAM findings are obtained

Type	F = 1	F = 2	F = 3	F = 4	F = 5	Avg
Traditional U-Net	74.11	72.67	73.89	75.22	76.07	74.392
Only encoder-decoder path	76.34	73.78	78.43	75.13	78.21	76.378
Only encoder-decoder convolution block	83.76	77.91	83.32	81.11	82.36	81.692
MDU-CNN	82.25	79.43	83.16	81.02	85.08	82.188

### Ablation study

**Fig. 11:**
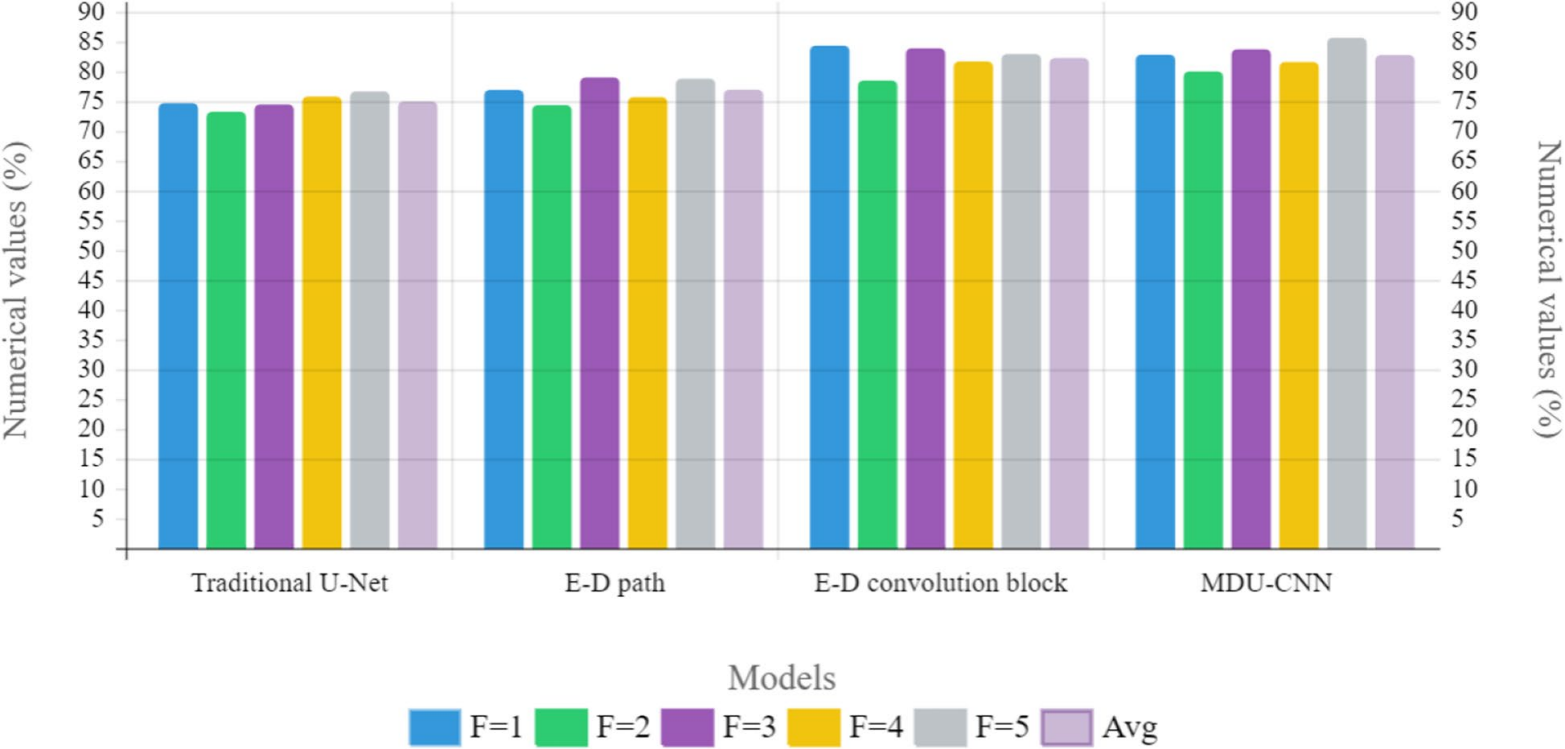
Graphical illustration of k-fold cross validation of different models from EndoSLAM

Ablation research was carried out to evaluate the individual contributions of the MultiDimensional blocks and the encoder-decoder convolution routes. The tests were carried out from two perspectives: in the first, the encoder-decoder convolution paths were simply inserted in a simple U-net based method for our first implementation and replaced one of its two convolutional blocks by using multi-dimensional ones for our second. With the aim of finding the best performing architecture, a comparison among multiple U-net models including those with encoder-decoder convolution routes, multi-dimensional blocks, and MDU-CNN was carried out. The rationale behind selecting the CVC - EndoSLAM dataset for this ablation study is that it proved to be more challenging than any other datasets we have used before. Additionally, findings from a test involving five folds for cross-validation will be shown on table number five. The addition of encoder-decoder convolution routes clearly outperforms the traditional U-Net, as seen in the table. The table clearly shows that the efficiency gains from using multidimensional blocks are considerably greater when they are used independently of an encoder-decoder link. The suggested MDU-CNN, which makes use of both encoder-decoder routes and multidimensional blocks, achieves its best results due to the complementary nature of these two features. Figure 11, depicts the different k-fold cross validation outcome of different models for EndoSLAM dataset.

### Comment on Data Augmentation (DA)

It is a common understanding that CNNs perform better when they are given a larger dataset. However, up until this point during the presentation all of these results have been obtained without adding extra data. The objective is to assess the general efficiency of MDU-CNN and traditional U-net by evaluating their abilities under similar conditions. Without more data available for training purposes, we have concluded that both models will prove challenging. An added hindrance that needs to be overcome is our initial analysis focused on certain models which incorporated data augmentation. As a result of its proven complexity, we resorted back to working with the EndoSLAM dataset. Our amount of training data was boosted by up to three times due to random flips and rotations or a combination thereof. In assessing their performance, we compared how well both models were able to perform. Table 6 provides a comparison of the outcomes obtained with and without DE.

**Table 6:** Data augmenting both models. 5-fold cross-validation yields EndoSLAM results

Type	F = 1	F = 2	F = 3	F = 4	F = 5	Avg
**Without DA**						
Traditional U-Net	74.11	72.67	73.89	75.22	76.07	74.392
MDU-CNN	82.25	79.43	83.16	81.02	85.08	82.188
**With DA**						
Traditional U-Net	81.76	77.91	81.32	78.11	79.36	79.692
MDU-CNN	85.25	84.03	88.12	84.02	85.08	85.3

**Fig. 12:**
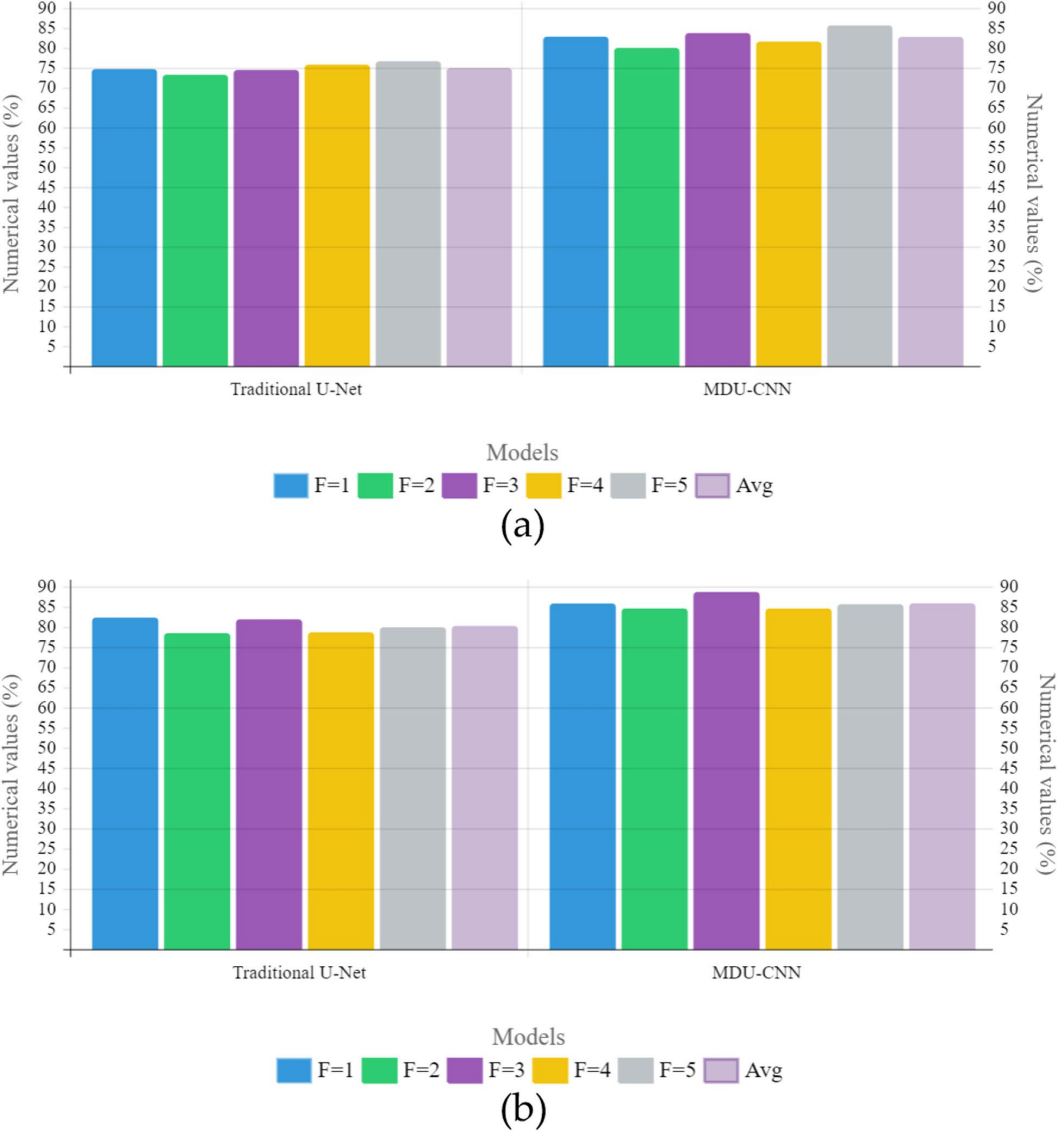
Graphical illustration of k-fold cross validation of different models from EndoSLAM **a** without data augmentation; **b** with augmentation

According to the experimental results, both models perform better with data augmentation, and MDU-CNN outperforms standard U-Net. MDU-CNN showed an increase of 3% whereas the improvement of Baseline U-NET model was slightly bigger with 5.31% is the score achieved by the U-net model with a low base line performance scoring only at 74.3%, to fall behind when compared with its counterpart MDU-CNN on this dataset; however increasing data through augmentation has provided some recognition for patterns once learnt easier by counterpart reaching a higher score (82.1%) and in Fig. 12 there is a comparison of the outcomes obtained using different models for EndoSLAM dataset under different conditions of data augmentation through k-fold cross validation.

## Conclusion

In the proposed work, we started by looking closely at the U-Net architecture to see if there were any ways it could be made better. We had assumed that there would be a discrepancy between the features that the encoder model shared and those that the decoder network subsequently propagated. We made encoder-decoder convolution paths, which use some pre-processing techniques to make the mappings of two features more similar to each other. We also proposed multidimensional blocks to add multi-resolution analysis capability to U-Net. We were inspired by the blocks in Inception, and as a result, we were able to design a compact equivalent framework that was lighter and required lesser memory. We proposed a new architecture, the MDU-convolutional neural network, by integrating these improvements. We chose the biomedical image datasets that were extremely distinct from one another and that were made available to the general public. Furthermore, each of those databases has its own distinct difficulty. The cell nuclei in the foreground and the rest of the image in the background are very different in the Murphy Laboratory Fluorescent Microscope database. This could make it the easiest dataset to use for segmentation. In the colon endoscopic images included in the EndoSLAM dataset, distinguishing polyps from the background is typically challenging even for a professional operator. This is a hard dataset to work with because the polyps are so different in shape, size, structure, direction, etc. However, the dermoscopy database that is part of the ISIC2020 challenge includes images that have low contrast to the degree that the skin lesions and the backdrop can often be confused for one another, and vice versa. Both foreground and background textures of varying types, complicates further pattern detection.

ISBI2022's electron microscopy data collection brings a new type of difficulty. As the region being segmented takes up most of the image in this dataset, there is a tendency for the images to be over-segmented. However, the BraTS20 MRI data comprises multimodal 3-dimensional images, which presents a unique challenge. Images should be as close to perfect as possible; segmentation is a challenging task, yet U-Net achieves impressive results. When compared to U-Net, our proposed framework just marginally outperforms it when it comes to these cases. However, the performance improvement by MDU-CNN is significantly greater for complex images that contain things like; noise, perturbations, unclear borders, etc. More precisely, MDU-CNN outperformed U-Net by a factor of 1.32%, 5.19%, 4.50%, 10.23%, and 0.87% across the five datasets shown in Table 4. As a result of these improvements, MDU-CNN segmentations not only score better on the evaluation criteria, but they also look closer to the ground truth. Further, U-Net appeared to oversegment, undersegment, make incorrect predictions, and even overlook the items entirely on the very difficult images. Actually, experimental results showed that MDU-CNN was more consistent and robust. MDU-CNN was capable of recognizing even the smallest subtle of image boundaries; it was durable in segmenting the images with a number of disruptions; and it was removable for outliers. While the U-Net had a tendency to oversegment the majority class, MDU-CNN was able to accurately classify the details. The 3-dimensional version of MDU-CNN also outperformed the 3-dimensional U-Net, which is more than just a 3-dimensional translation of the 2-dimensional U-Net; it provides other improvements and enhancements. To be fair, the proposed MDU-CNN did not always produce perfect segmentations, although it did far better than the standard U-Net. Hence, we agree our proposed MDU-CNN framework can be the possible replacement to the traditional U-Net design.

## Future work

In the proposed work, we tried to keep our model's number of parameters about the same as the U-Net model's. In the future, we want to do more tests to identify the best way to combine the method's hyper-parameters. In the near future, we also plan to test our framework on clinical images taken with different methods. In addition to this, we plan to conduct experiments in which our model is subjected to a variety of pre and post processing approaches that are domain and implementation-relevant. As such, we expect that connecting our approach to a domain-specific, professional expertise-based pipeline and combining it with appropriate post-processing steps would further enhance our model's effectiveness and enable us all to design efficient segmentation approaches for a variety of applications. The successful implementation of Multimodal Biomedical Image Segmentation using a Multi-Dimensional U-Convolutional Neural Network not only advances the field of medical imaging but also paves the way for practical applications with significant implications for diagnosis, treatment, and overall healthcare delivery.

## Data Availability

The datasets used during the current study are available from the corresponding author on reasonable request.

## Abbreviations

MDU-CNN: MultiDimensional—Convolutional Neural Network
CAD: Computer-Aided Diagnostic
DL: Deep Learning
ISBI: International Symposium on Biomedical Imaging
U-Net: U-shaped -encode and decode network
LBCDN: Local Binarization Convolutional Neural Network
DC-U-net: Dual Channel Effect U-shaped -encode and decode network
ECA-Net: Efficient Channel Attention
RES-U-NET: Deep Residual UNET U-shaped -encode and decode network
VGG-16UNET: Visual Geometry Group 16
DENSENET: Densely Connected Convolutional Networks
ISIC: International Skin Imaging Collaboration
ReLU: Rectified Linear Unit
2D: 2-Dimensional
3D: 3-Dimensional
Ph2: Pedro Hispano
HGG: High-Grade Gliomas
LGG: Low-Grade Gliomas
MRI: Magnetic Resonance Image
BraTS2020: Multimodal Brain Tumor Segmentation Challenge 2020
HAM-10000: Human Against Machine with 10000
U2OS: Human Bone Osteosarcoma Epithelial Cells
EndoSLAM: Endoscopic Simulta- Neous Localization and Mapping
ssTEM: Transmission Electron-Microscopy
RAM: Random Access Memory
JI: Jaccard index
